# ChatGPT in healthcare: perceptions, ethical considerations, and practice implications among healthcare professionals in Ecuador and other countries in the Americas: a cross-sectional survey study

**DOI:** 10.3389/fdgth.2026.1730508

**Published:** 2026-06-10

**Authors:** Ivan Cherrez-Ojeda, Juan Carlos Gallardo-Bastidas, Marco Faytong-Haro, Freya Andrade Vera, Wael Hafez, Zouina Sarfraz, Azza Sarfraz, Juan Carlos Calderón, Karla Robles-Velasco, Johannes Eschrich

**Affiliations:** 1 Institute for Allergology, Charité – Universitätsmedizin Berlin, Corporate Member of Freie Universität Berlin and Humboldt-Universität zu Berlin, Berlin, Germany; 2 Fraunhofer Institute for Translational Medicine and Pharmacology ITMP, Immunology and Allergology, Berlin, Germany; 3 Universidad Espiritu Santo, Samborondon, Ecuador; 4 Respiralab Research Group, Guayaquil, Ecuador; 5 School of Dentistry, Universidad Católica Santiago de Guayaquil, Guayaquil, Ecuador; 6 Facultad de Investigación, Universidad Estatal de Milagro, Milagro, Guayas, Ecuador; 7 Instituto de Investigación, Universidad Agraria del Ecuador, Guayaquil, Ecuador; 8 Universidad Laica Eloy Alfaro de Manabí, Manta, Ecuador; 9 NMC Royal Hospital, Khalifa City, Abu Dhabi, United Arab Emirates; 10 Medical Research and Clinical Studies Institute, The National Research Centre, Cairo, Egypt; 11 Department of Medicine, Fatima Jinnah Medical University, Lahore, Pakistan; 12 Department of Pediatrics and Child Health, Aga Khan University, Karachi, Pakistan; 13 Department of Hepatology and Gastroenterology, Charité – Universitätsmedizin Berlin, Berlin, Germany

**Keywords:** artificial intelligence, ChatGPT, digital health, ethics, healthcare professionals, Latin America, professional attitudes

## Abstract

**Background:**

Generative artificial intelligence tools, such as ChatGPT, are increasingly discussed in healthcare; however, evidence from Latin American professional settings is limited and must be interpreted in light of regional digital inequities and ethical concerns.

**Objective:**

To examine awareness, ethical perceptions, usage patterns, and attitude determinants related to ChatGPT among HCPs practicing mainly in Ecuador and other countries in the Americas.

**Methods:**

We conducted a cross-sectional online survey between May and September 2023. Recruitment was conducted using professional networks, institutions, conferences, and social media. Descriptive analyses were performed for the entire sample. Ethical and usefulness analyses were restricted to respondents with at least minimal knowledge of ChatGPT (*n* = 424), and models involving job facilitation, perceived risk, and future intention were restricted to prior users with complete data (*n* = 218–230, depending on the model). Adjusted linear and binary logistic regression models controlled for age, gender, professional group, years of experience, and country (Ecuador vs. other).

**Results:**

Of the 747 respondents, 610 (81.66%) practiced in Ecuador, 281 (37.62%) were physicians, 203 (27.18%) were dentists, and 42 (5.62%) were nurses or allied health professionals. Overall, 424 (56.76%) had heard of ChatGPT, 239 (31.99%) had ever used it, and 323 (43.24%) reported no knowledge of it. Among respondents with at least minimal familiarity, 311/394 (78.93%) agreed that ChatGPT could be beneficial in healthcare settings, 256/389 (65.81%) considered it trustworthy for healthcare information, and 286/391 (73.15%) considered it useful for specific medical questions. Ethical judgments were mixed: 179/395 (45.32%) were neutral about language revision in a scientific manuscript, 189/395 (47.85%) considered AI-generated manuscript text unethical, and 268/395 (67.85%) considered sole-source clinical use unethical. Among prior users, the most common uses were homework or academic support (134/239, 56.07%), research writing support (117/239, 48.95%), and medical or healthcare education and training (55/239, 23.01%). Higher knowledge and ethical acceptance scores were independently associated with more favorable attitudes. Ethical acceptance predicted stronger agreement that ChatGPT could be beneficial in healthcare settings (*β* = 0.30, 95% CI 0.19 to 0.41) and trustworthy (*β* = 0.32, 95% CI 0.20 to 0.44). Future weekly-or-more use was more likely among respondents who viewed ChatGPT as beneficial in healthcare (adjusted OR 1.95, 95% CI 1.39 to 2.76) and useful for specific medical questions (adjusted OR 1.97, 95% CI 1.46 to 2.65).

**Conclusions:**

Healthcare professionals in this sample showed cautious optimism toward ChatGPT, but awareness remained moderate, use was limited, and ethical reservations were substantial. Structured, context-specific training and more regionally representative studies are needed before large-scale integration can be recommended.

## 1 Introduction

Artificial intelligence (AI) and large language models are increasingly influencing clinical care, patient communication, education, and research. In healthcare, these systems are being used or tested for information synthesis, decision support, documentation, patient triage, and mental health communication, among other tasks (1–5). ChatGPT has become the most visible example of this shift, largely because it offers conversational access to capabilities that were previously confined to more technical AI environments (2–5).

The rapid diffusion of ChatGPT has also raised substantial ethical questions. In healthcare, the main concerns extend beyond simple usefulness and include data privacy, data security, patient confidentiality, hallucinated or misleading outputs, algorithmic bias, authorship transparency, and accountability for clinical decisions (6–9). These issues are especially salient when AI-generated text enters patient-facing communication, medical documentation, or scientific writing, where errors can affect both patient safety and professional integrity.

The Latin American context adds another layer of complexity. PAHO and regional policy reports have documented major differences across countries in digital infrastructure, interoperability, information systems, workforce digital literacy, and institutional readiness for digital health transformation (10–13). These structural differences matter because they can shape not only who has access to tools such as ChatGPT but also whether those tools are institutionally governed or used informally by individual professionals.

Despite growing global interest in generative AI, most published evidence on ChatGPT in health-related settings has focused on students, single specialties, or samples drawn mainly from high-income countries (14–18). Data on practicing professionals in Latin America remain limited, and the region has particular relevance because of its simultaneous expansion of digital health initiatives and persistent socioeconomic and digital inequities (10, 11, 19).

Accordingly, this study aimed to assess awareness, ethical perceptions, attitudes, further learning interests, and use patterns related to ChatGPT among HCPs who practice mainly in Ecuador and other countries in the Americas. The study was guided by the following research question: What knowledge levels, ethical perceptions, and attitudinal factors are associated with ChatGPT adoption among HCPs in this regional context?

## 2 Materials and methods

### 2.1 Design and ethics approval

This cross-sectional web-based survey was conducted from May to September 2023. Ethical approval was obtained from the Comité de Ética en Investigación en Seres Humanos (CEISH), Hospital Clínica Kennedy, Guayaquil, Ecuador. All participants provided electronic informed consent before accessing the questionnaire.

The survey was administered through an anonymous Qualtrics web link that was distributed via email, professional networks, healthcare associations, conferences, and social media. No incentives were offered.

### 2.2 Participants, recruitment, and analytic sample

Eligible respondents were healthcare professionals aged 18 years or older who were practicing in Ecuador or other countries in the Americas and who completed the online survey. Recruitment used convenience sampling and was designed to reach a wide range of professional backgrounds, including physicians, dentists, nurses, and other health workers.

The initial dataset contained 1,084 survey records. After excluding respondents who did not provide consent, those with insufficient information to classify ChatGPT knowledge, records without a usable country designation, and responses from outside the Americas, 747 records remained in the final analytical sample.

Although the study was conceived for Latin American recruitment, the open-link design also yielded a small number of responses from elsewhere in the Americas, mainly the United States and Canada. These observations were retained for descriptive analyses because they were collected through the same professional dissemination channels and represented a small minority of the sample. In regression analyses, country was therefore collapsed as Ecuador versus other because cell sizes outside Ecuador were small.

Using a population benchmark of approximately 2 million health professionals in Latin America and the Caribbean, the achieved sample size corresponds to an illustrative margin of error of approximately ±3.6% at the 95% confidence level under simple random sampling assumptions. Because the present study used convenience sampling, this estimate is presented only as a measure of descriptive precision and not as a claim of representativeness (20).

### 2.3 Questionnaire and study variables

The questionnaire was adapted from a previously published survey used among healthcare students and was structured in accordance with established guidance for web-based survey reporting (15, 21). Before field deployment, the wording was reviewed by the study team and piloted with healthcare professionals to improve contextual clarity for Latin American practice settings.

The instrument included demographic and professional items, ChatGPT familiarity and self-perceived knowledge, ethical judgments, beliefs about perceived risk, use patterns, attitudes, and further learning needs. For clarity in the revised analyses, professional status was collapsed into four broad groups: physicians, dentists, nurses and allied health professionals, and other healthcare professionals.

Self-perceived knowledge of ChatGPT was classified into three categories: no knowledge, minimal knowledge, and adequate knowledge. The ethics domain included three items that asked respondents how ethical they considered ChatGPT to be for revising the language of a scientific manuscript, writing text within a scientific manuscript, and serving as the sole information source for clinical practice.

The perceived-risk domain comprised three statements: whether AI could replace the respondent's job in the future, whether ChatGPT or similar technologies would play a more important role in the respondent's work, and whether using AI like ChatGPT in clinical practice raises ethical concerns. The attitude domain included five statements assessing whether ChatGPT makes work easier, can be beneficial in healthcare settings, provides trustworthy healthcare information or guidance, is useful for specific medical questions, and is useful for searching medical literature.

Pattern-of-use items assessed whether respondents had ever used ChatGPT, their expected future frequency of use, and the applications for which they used it, including academic support, research writing, medical education, health communication, clinical decision support, electronic health record documentation, patient triage, and mental health support. Further-learning items asked respondents whether they wanted additional training and which topics they considered most important, including specific medical use cases, data privacy and security, and ethical considerations.

### 2.4 Data-quality procedures

Because the survey link was anonymous and distributed across multiple channels, platform-level single-response enforcement could not be guaranteed. Therefore, we conducted *post hoc* quality checks based on response ID, IP address, timestamps, completion time, and exact-response patterns.

Repeated IP addresses were common, which was expected because professionals may have responded from shared institutional networks. One exact duplicate response pattern from the same IP address was identified. Excluding one of the two records did not materially change the descriptive estimates; therefore, the full eligible sample was retained for the main analyses. Item-level missingness within completed questionnaire branches was minimal because relevant items were mandatory, although structurally missing data were generated by survey skip logic.

### 2.5 Statistical analysis

Descriptive statistics were calculated for the entire sample and for the relevant analytical subsets. Continuous variables are reported as means with standard deviations or medians with interquartile ranges, and categorical variables are reported as counts and percentages with two decimal places.

Attitudinal and ethical analyses were restricted to participants with at least minimal knowledge of ChatGPT (*n* = 424). Items related to job facilitation, perceived risk, and future intention were available only to respondents who had previously used ChatGPT; accordingly, these analyses were based on smaller complete-case subsets (*n* = 218–230, depending on the model).

Two adjusted linear regression models examined whether years of professional experience were associated with the perceived-risk and ethical-acceptance composite scores. These models included years of experience as the main predictor and controlled for gender, professional group, and country. Age was examined separately in the sensitivity models rather than entered simultaneously with years of experience because the two measures captured overlapping constructs and introduced collinearity.

A second set of adjusted linear regression models examined whether knowledge scores, perceived-risk scores, or ethical-acceptance scores predicted each of the five attitude items. In these models, the dependent variables were the five attitude statements, and the independent variables of interest were entered one at a time. All models were adjusted for age, gender, professional group, years of experience, and country.

A final set of adjusted binary logistic regression models examined whether favorable attitudes predicted the intention to use ChatGPT at least weekly over the next 6 months versus monthly or less. Each attitude item was entered as the main independent variable in a separate model with the same covariate set as above.

Linear models were estimated using heteroscedasticity-robust HC3 standard errors. Multicollinearity was assessed using variance inflation factors, which remained low in the final models (maximum VIF <2.3 in the attitude models and <2.1 in the experience models). Composite scores and model residuals were inspected descriptively because the outcomes were based on bounded Likert-type items. Analyses were conducted in Python 3.13 using pandas 2.2.3 and statsmodels 0.14.6. Statistical significance was defined as *P* < 0.05.

## 3 Results

### 3.1 Sample characteristics

Of the total sample (*n* = 747), only participants with at least minimal knowledge of ChatGPT (*n* = 424) were included in attitudinal and ethical analyses. Sample sizes therefore vary across analyses due to item non-response and model-specific missing data. Table 1 summarizes the full analytical sample (*N* = 747). The mean age was 35.94 years (SD = 14.57), 432 respondents (57.83%) were women, and 518 respondents (69.35%) belonged to Generation Z or the Millennial generation. A total of 610 respondents (81.66%) practiced in Ecuador. Smaller groups practiced in the United States (*n* = 41, 5.49%), Colombia (*n* = 31, 4.15%), Mexico (*n* = 20, 2.68%), Argentina (*n* = 14, 1.87%), the Dominican Republic (*n* = 12, 1.61%), and Brazil (*n* = 8, 1.07%), with the remaining countries contributing fewer than five participants each.

**Table 1:** Sociodemographic and professional characteristics of the full analytic sample (*N* = 747).

Characteristic	*N*	%
Age, mean (SD)	35.94 (14.57)	
Generation Z	258	34.54
Millennial	260	34.81
Generation X	149	19.95
Baby Boomer	80	10.71
Male	308	41.23
Female	432	57.83
Prefer not to say	1	0.13
Other	6	0.80
Physicians	281	37.62
Dentists	203	27.18
Nurses and allied health	42	5.62
Other healthcare professionals	221	29.59
≤5 years	210	28.11
>5 years	537	71.89
Ecuador	610	81.66
Other countries in the Americas	137	18.34
Heard of ChatGPT	424	56.76
No prior awareness of ChatGPT	323	43.24
Ever used ChatGPT	239	31.99
Self-perceived knowledge: No knowledge	323	43.24
Self-perceived knowledge: Minimal knowledge	172	23.03
Self-perceived knowledge: Adequate knowledge	252	33.73

Percentages are based on the full analytic sample. Professional roles were collapsed into four broad categories for interpretability.

Professional roles were regrouped into four broad categories to improve interpretability: physicians (281/747, 37.62%), dentists (203/747, 27.18%), nurses and allied health professionals (42/747, 5.62%), and other healthcare professionals (221/747, 29.59%). Table 2 presents the distribution of these broad professional groups across countries and confirms that Ecuadorian respondents predominated in every category. The relatively large dentist subgroup likely reflects recruitment-channel effects related to academic and professional networks rather than the underlying composition of the regional healthcare workforce.

**Table 2:** Distribution of participants by country of practice and professional group.

Country	Total	% of sample	Physicians	Dentists	Nurses and allied health	Other healthcare professionals
Argentina	14	1.87	10	1	0	3
Brazil	8	1.07	2	0	0	6
Canada	1	0.13	1	0	0	0
Chile	4	0.54	1	2	0	1
Colombia	31	4.15	4	23	1	3
Costa Rica	2	0.27	1	0	0	1
Dominican Republic	12	1.61	9	0	0	3
Ecuador	610	81.66	208	176	38	188
Honduras	1	0.13	0	0	0	1
Mexico	20	2.68	15	0	2	3
Paraguay	1	0.13	1	0	0	0
Peru	1	0.13	0	0	0	1
Puerto Rico	1	0.13	1	0	0	0
USA	41	5.49	28	1	1	11
Total	747	100.00	281	203	42	221

Counts are shown by country and by the four broad professional groups used in the revised analysis. Because most countries outside Ecuador had small cell sizes, regression models collapsed country as Ecuador versus other.

Awareness of ChatGPT was moderate, but usage was limited. Overall, 424 respondents (56.76%) had heard of ChatGPT, 239 (31.99%) had ever used it, and 323 (43.24%) reported no knowledge of it. Self-perceived knowledge was classified as minimal in 172 respondents (23.03%) and adequate in 252 respondents (33.73%).

Because 323 participants reported no prior knowledge of ChatGPT, the attitudinal and ethical analyses reported below should be interpreted as reflecting respondents with at least minimal familiarity (*n* = 424) rather than the entire sample. All subsequent analyses involving perceptions, attitudes, and ethics are restricted to participants with prior knowledge of ChatGPT (*n* = 424). In addition, items on job facilitation, perceived risk, and future-use intention were answered only by prior users; therefore, the corresponding analyses represent an early-adopter subgroup (*n* = 239, with complete-case denominators ranging from 218 to 230 depending on the model).

### 3.2 Ethics, attitudes, use patterns, and learning needs

Table 3 summarizes the descriptive results for awareness, ethics, attitudes, use patterns, and training interests across the relevant analytical subsets. Among respondents with at least minimal knowledge of ChatGPT, the median overall attitude score was 4.00 (IQR: 3.40–4.40), indicating generally favorable views. The median ethical acceptance score was 2.33 (IQR: 2.00–3.00), reflecting a more cautious stance. Among prior users, the median perceived-risk score was 3.33 (IQR: 2.67–3.67).

**Table 3:** ChatGPT awareness, ethics, attitudes, use, and training interests across the relevant analytic subsets.

Measure	*n*/*N*	%
Respondents with at least minimal ChatGPT knowledge	424	56.76
Respondents who had ever used ChatGPT	239	31.99
Agreed that ChatGPT can be beneficial in healthcare settings (among valid responses)	311/394	78.93
Agreed that ChatGPT provides trustworthy healthcare information or guidance (among valid responses)	256/389	65.81
Agreed that ChatGPT is useful for specific medical questions (among valid responses)	286/391	73.15
Agreed that ChatGPT is useful for searching medical literature (among valid responses)	287/387	74.16
Agreed that ChatGPT makes my job easier (among valid responses)	118/224	52.68
Considered language revision in a scientific manuscript unethical (among valid responses)	105/395	26.58
Considered writing manuscript text unethical (among valid responses)	189/395	47.85
Considered sole-source clinical use unethical (among valid responses)	268/395	67.85
Used ChatGPT for homework or academic support (among ever users)	134/239	56.07
Used ChatGPT for research writing support (among ever users)	117/239	48.95
Used ChatGPT for medical/healthcare education and training (among ever users)	55/239	23.01
Wanted to learn more about ChatGPT (among valid responses)	600/703	85.35
Interested in data privacy and security measures (among respondents wanting further learning)	249/600	41.50
Interested in ethical considerations (among respondents wanting further learning)	240/600	40.00
Interested in specific medical use cases (among respondents wanting further learning)	389/600	64.83

Denominators vary because of survey branching and item-specific complete cases. Ethics and usefulness analyses were restricted to respondents with at least minimal knowledge of ChatGPT, and some later items were available only to prior users.

Positive attitudes toward usefulness were common. Of the valid responses, 311 out of 394 respondents (78.93%) agreed that ChatGPT can be beneficial in healthcare settings, 256 out of 389 (65.81%) agreed that it provides trustworthy healthcare information or guidance, 286 out of 391 (73.15%) agreed that it is useful for specific medical questions, and 287 out of 387 (74.16%) agreed that it is useful for searching medical literature. Agreement that ChatGPT makes work easier was lower and was reported by 118 out of 224 prior users (52.68%).

Ethical judgments varied by use case. Revising the language of a scientific manuscript was most often judged as neither ethical nor unethical (179/395, 45.32%). By contrast, 189 of 395 respondents (47.85%) considered writing text within a scientific manuscript unethical, and 268 of 395 (67.85%) considered using ChatGPT as the sole source of information for clinical practice unethical.

Risk-related beliefs also showed a nuanced profile. Most prior users disagreed that AI could replace their jobs in the future (126/225, 56.00%). At the same time, 136 of 225 (60.44%) agreed that ChatGPT or similar technologies will play an even more important role in their work, and 133 of 223 (59.64%) agreed that using AI like ChatGPT in clinical practice raises ethical concerns.

Among respondents who had used ChatGPT, the most common applications were homework or academic support (134/239, 56.07%), research writing support (117/239, 48.95%), and medical or healthcare education and training (55/239, 23.01%). Health communication (39/239, 16.32%) and electronic health record documentation (32/239, 13.39%) were less common, and patient triage was uncommon (17/239, 7.11%).

The demand for additional training was substantial. Of the 703 respondents who provided a valid answer to the training-interest item, 600 (85.35%) wanted to learn more about ChatGPT. Among those interested in further learning, the most frequently selected topics were specific medical use cases (389/600, 64.83%), data privacy and security measures (249/600, 41.50%), and ethical considerations (240/600, 40.00%). Taken together, these findings suggest cautious optimism among early adopters rather than population-wide acceptance.

### 3.3 Experience-related differences in perceived risk and ethical acceptance

Table 4 presents the adjusted models for experience-related differences. Compared with professionals with five years of experience or less, those with more than 10 years of experience had lower perceived-risk scores (*β* = −0.34, 95% CI −0.63 to −0.05; *P* = 0.021) and lower ethical-acceptance scores (*β* = −0.27, 95% CI −0.49 to −0.05; *P* = 0.015) after adjusting for sex, professional group, and country.

**Table 4:** Adjusted linear regression models comparing perceived-risk and ethical-acceptance scores across years of professional experience.

Outcome	Predictor	Adjusted *β* (95% CI)	P	Adjusted R^2^	Max VIF	*N*
Perceived risk score	6–10 years vs ≤5 years	−0.01 (−0.38 to 0.35)	0.935	0.028	2.05	225
Perceived risk score	>10 years vs ≤5 years	−0.34 (−0.63 to −0.05)	0.021	0.028	2.05	225
Ethical acceptance score	6–10 years vs ≤5 years	−0.21 (−0.48 to 0.05)	0.115	0.004	1.85	395
Ethical acceptance score	>10 years vs ≤5 years	−0.27 (−0.49 to −0.05)	0.015	0.004	1.85	395

Reference group: ≤5 years of professional experience. Models were adjusted for gender, professional group, and country. Age was examined separately in sensitivity analyses because of collinearity with years of experience.

No statistically significant differences were observed in the 6–10 year group. Separate age-based sensitivity models did not show independent age associations once the same covariates were considered, which supported the decision to present the experience-based models as the main results.

### 3.4 Knowledge, perceived risk, and ethical acceptance as predictors of favorable attitudes

Table 5 presents the adjusted linear regression models. Higher knowledge scores were associated with stronger agreement that ChatGPT could be beneficial in healthcare settings (*β* = 0.18, 95% CI 0.09 to 0.27; *P* < 0.001), provide trustworthy healthcare information or guidance (*β* = 0.12, 95% CI 0.00 to 0.23; *P* = 0.049), and be useful for specific medical questions (*β* = 0.12, 95% CI 0.01 to 0.23; *P* = 0.033). Knowledge was not significantly associated with the job-facilitation item or the medical-literature item in the adjusted models.

**Table 5:** Adjusted linear regression models of knowledge, perceived-risk, and ethical-acceptance scores predicting ChatGPT attitudes.

Predictor	Outcome	Adjusted β (95% CI)	P	Adjusted R^2^	Max VIF	*N*
Knowledge score	ChatGPT makes my job easier	0.02 (−0.16 to 0.19)	0.827	0.023	2.23	224
Knowledge score	ChatGPT can be beneficial in healthcare settings	0.18 (0.09 to 0.27)	<0.001	0.048	2.14	394
Knowledge score	ChatGPT provides trustworthy healthcare information or guidance	0.12 (0.00 to 0.23)	0.049	0.050	2.15	389
Knowledge score	ChatGPT is useful for specific medical questions	0.12 (0.01 to 0.23)	0.033	0.013	2.14	391
Knowledge score	ChatGPT is useful for searching medical literature	0.03 (−0.09 to 0.15)	0.613	0.010	2.13	387
Perceived risk score	ChatGPT makes my job easier	0.50 (0.33 to 0.68)	<0.001	0.139	2.25	224
Perceived risk score	ChatGPT can be beneficial in healthcare settings	0.08 (−0.11 to 0.26)	0.430	0.054	2.26	225
Perceived risk score	ChatGPT provides trustworthy healthcare information or guidance	0.15 (−0.04 to 0.34)	0.125	0.043	2.25	222
Perceived risk score	ChatGPT is useful for specific medical questions	0.19 (−0.00 to 0.38)	0.051	0.021	2.26	225
Perceived risk score	ChatGPT is useful for searching medical literature	0.19 (0.00 to 0.38)	0.049	0.046	2.25	222
Ethical acceptance score	ChatGPT makes my job easier	0.49 (0.28 to 0.70)	<0.001	0.124	2.25	224
Ethical acceptance score	ChatGPT can be beneficial in healthcare settings	0.30 (0.19 to 0.41)	<0.001	0.087	2.15	394
Ethical acceptance score	ChatGPT provides trustworthy healthcare information or guidance	0.32 (0.20 to 0.44)	<0.001	0.104	2.16	389
Ethical acceptance score	ChatGPT is useful for specific medical questions	0.30 (0.17 to 0.42)	<0.001	0.058	2.16	391
Ethical acceptance score	ChatGPT is useful for searching medical literature	0.26 (0.13 to 0.39)	<0.001	0.051	2.15	387

Each row represents a separate adjusted linear regression model. Models adjusted for age, gender, professional group, years of experience, and country (Ecuador vs other). HC3 robust standard errors were used.

Ethical acceptance showed the most consistent pattern. Higher ethical acceptance scores were independently associated with more favorable responses for every attitude item, including job facilitation (*β* = 0.49, 95% CI 0.28 to 0.70; *P* < 0.001), perceived benefit in healthcare settings (*β* = 0.30, 95% CI 0.19 to 0.41; *P* < 0.001), trustworthiness (*β* = 0.32, 95% CI 0.20 to 0.44; *P* < 0.001), usefulness for specific medical questions (*β* = 0.30, 95% CI 0.17 to 0.42; *P* < 0.001), and usefulness for medical literature searches (*β* = 0.26, 95% CI 0.13 to 0.39; *P* < 0.001).

The perceived-risk composite showed a more selective pattern. Higher scores were associated with stronger agreement that ChatGPT makes work easier (*β* = 0.50, 95% CI 0.33 to 0.68; *P* < 0.001) and that it is useful for searching medical literature (*β* = 0.19, 95% CI 0.00 to 0.38; *P* = 0.049), while associations with the other attitude items were weaker or not statistically significant.

Model fit was modest but stable, with adjusted R^2^ values ranging from 0.010 to 0.139. Multicollinearity was low in all models. In sensitivity analyses excluding Ecuadorian respondents, the direction of the key knowledge- and ethics-related associations remained unchanged, although the confidence intervals widened because of the smaller sample size.

### 3.5 Future intention to use ChatGPT

Among prior users, more favorable attitudes were consistently associated with a greater likelihood of intending to use ChatGPT at least weekly during the next 6 months (Table 6). Respondents who agreed that ChatGPT makes their job easier had higher adjusted odds of frequent future use (adjusted OR 1.62, 95% CI 1.26 to 2.09; *P* < 0.001). Similar associations were observed for believing that ChatGPT can be beneficial in healthcare settings (adjusted OR 1.95, 95% CI 1.39 to 2.76; *P* < 0.001), provides trustworthy information (adjusted OR 1.56, 95% CI 1.18 to 2.06; *P* = 0.002), is useful for specific medical questions (adjusted OR 1.97, 95% CI 1.46 to 2.65; *P* < 0.001), and is useful for searching medical literature (adjusted OR 1.52, 95% CI 1.18 to 1.97; *P* = 0.001).

**Table 6:** Adjusted binary logistic regression models predicting intention to use ChatGPT at least weekly during the next 6 months among prior users.

Predictor	Adjusted OR (95% CI)	P	Nagelkerke R^2^	*N*
ChatGPT makes my job easier	1.62 (1.26 to 2.09)	<0.001	0.131	224
ChatGPT can be beneficial in healthcare settings	1.95 (1.39 to 2.76)	<0.001	0.131	230
ChatGPT provides trustworthy healthcare information or guidance	1.56 (1.18 to 2.06)	0.002	0.094	227
ChatGPT is useful for specific medical questions	1.97 (1.46 to 2.65)	<0.001	0.161	230
ChatGPT is useful for searching medical literature	1.52 (1.18 to 1.97)	0.001	0.099	227

Each row represents a separate adjusted logistic regression model comparing anticipated use at least weekly versus monthly or less. Models adjusted for age, gender, professional group, years of experience, and country (Ecuador vs other).

Nagelkerke R^2^ values ranged from 0.094 to 0.161, indicating modest explanatory power. These findings support the interpretation that perceived usefulness and trust are central drivers of continued adoption among respondents with some direct experience with the tool.

## 4 Discussion

This study provides one of the earliest multi-professional surveys of ChatGPT perceptions among HCPs practicing mainly in Ecuador and a smaller set of other countries in the Americas. Three findings are particularly important. First, awareness was only moderate, and actual use remained limited. Second, respondents clearly distinguished between relatively acceptable low-stakes uses and less acceptable high-stakes uses. Third, greater knowledge and stronger ethical acceptance were consistently associated with more favorable attitudes and higher intended future use. Because 81.66% of the sample was based in Ecuador, these findings should be interpreted primarily within the Ecuadorian context rather than as representative of the entire Latin American healthcare workforce.

Our estimate of ChatGPT use was lower than that reported in some specialty-specific or high-income settings. In the global urology survey by Eppler et al., almost half of the respondents reported academic use of ChatGPT-like tools, whereas only approximately one-third of our respondents had ever used ChatGPT (14). Temsah et al. and Hosseini et al. similarly described growing interest among healthcare workers and trainees; however, they also emphasized uneven hands-on experience, uncertainty about safe implementation, and concerns about reliability (16, 17). One plausible explanation for the lower uptake in our sample is that adoption still occurs largely through individual initiative rather than through formal institutional programs.

This interpretation is consistent with regional policy reports. PAHO and ECLAC have documented heterogeneous progress in digital transformation across the Americas, including uneven interoperability, connectivity, digital literacy, and health-information infrastructure (10–13). In such settings, generative AI tools are likely to enter professional practice first as informal productivity tools rather than as institutionally authorized clinical systems. This distinction matters because informal adoption often expands faster than governance, training, and accountability structures.

The ethical profile observed in this study was nuanced. Respondents were relatively tolerant of using ChatGPT to revise the language of a scientific manuscript but were much less accepting of using AI to write manuscript text and strongly rejected ChatGPT as a sole information source for clinical practice. This gradient is conceptually important because it separates editorial assistance from the substitution of professional judgment. It also aligns with broader ethical literature that highlights patient confidentiality, hallucinated or unverifiable information, disclosure of AI assistance, authorship integrity, and human responsibility for clinical decisions as central concerns in healthcare AI (6–9, 22).

Our findings on training needs reinforce this interpretation. Among respondents who wanted to learn more about ChatGPT, data privacy and security measures and ethical considerations were among the most frequently selected topics. Hu et al. reported similar enthusiasm for structured ChatGPT-assisted training among healthcare trainees and professionals while underscoring the need for expert guidance (18). Sarıkahya et al. likewise found that nursing faculty viewed ChatGPT as both an innovative educational resource and a challenge that required stronger ethical literacy and digital competence (23). Taken together, these findings suggest that training should extend beyond basic platform familiarity and explicitly address privacy, misinformation, documentation standards, authorship disclosure, and safe boundaries for clinical use.

The association between knowledge and favorable attitudes suggests that acceptance is not driven solely by novelty. Greater familiarity may help professionals identify realistic use cases while also recognizing where caution is needed. This interpretation is consistent with prior work showing that perceived usefulness, ease of integration, and trust are major determinants of AI acceptance in health settings (24–27). In our study, ethical acceptance was the strongest and most consistent attitudinal predictor, suggesting that professionals do not view usefulness and ethics as competing frames. Rather, they appear more willing to value the tool when they also believe that its use can be bounded by acceptable professional norms.

Experience-related findings also deserve careful interpretation. Professionals with > 10 years of experience had lower perceived-risk and ethical-acceptance scores than respondents with 5 years of experience or less. One possible explanation is that more established clinicians evaluate AI from the perspective of workflow stability, accountability, and prior experience with technological transitions. Previous research on clinical decision support systems has similarly shown that adoption depends not only on technical performance but also on workflow fit, institutional trust, and professional culture (9, 28). At the same time, most prior users in our sample expected AI tools to become more important in their work, which indicates that caution about unrestricted use coexists with recognition of likely long-term relevance.

International comparisons should be made carefully; however, the overall pattern is not unique. Surveys from China and Pakistan have also reported substantial interest in AI, along with important differences according to prior exposure and training opportunities (25, 26). Compared with a previously published student survey that used a closely related instrument, the professionals in our study appeared more conservative about manuscript authorship and sole-source clinical use, which is unsurprising given their direct responsibility for patient care and professional accountability (15).

From a policy perspective, the most practical implication is that healthcare institutions in the region need context-specific governance rather than either unrestricted enthusiasm or blanket prohibition. Institutional guidance should clarify acceptable uses, rules for patient-data handling, expectations for human verification of outputs, disclosure requirements for AI-assisted scholarly work, and the limits of using generative models in clinical decision-making (6, 9, 27). Such guidance is likely to be especially important in Latin American settings, where digital transformation is advancing but remains uneven across countries and institutions.

## 5 Strengths and limitations

This study has several limitations. First, the sample was not regionally representative. Ecuador accounted for 81.66% of all responses, and a small number of observations came from elsewhere in the Americas through the open survey link. Therefore, the results should not be generalized to the Latin American healthcare workforce as a whole.

Second, the convenience sampling strategy may have overrepresented professionals who were more digitally engaged or more interested in AI. The comparatively large dentist subgroup likely reflects recruitment-channel effects rather than the composition of the underlying professional population.

Third, several substantive analyses relied on smaller analytical subsets because respondents with no prior knowledge of ChatGPT did not complete later sections and some questions were asked only of prior users. The attitudinal and future-intention models therefore describe respondents with at least minimal familiarity and, in some cases, direct experience with the tool. This early-adopter profile may bias the results toward more favorable views.

Fourth, repeated IP addresses can not be treated automatically as invalid because institutional networks often share Internet addresses. Although *post hoc* screening identified only one pair of exact duplicate response patterns, and removing one record did not materially change the descriptive estimates, some residual duplication risk remains.

Fifth, the cross-sectional design and self-reported measures did not allow for causal inferences. Unmeasured organizational factors, such as local policies or institutional endorsements of AI tools, were not captured directly.

These limitations should be weighed against several strengths. The study included multiple professional groups, examined concrete ethical and practical use cases rather than generic opinions alone, and used adjusted models to clarify the relationship between knowledge, ethics, attitudes, and future intention. In addition, sensitivity analyses excluding Ecuadorian respondents preserved the direction of the key knowledge- and ethics-related associations, although the precision was reduced.

## 6 Conclusions

In this sample of healthcare professionals practicing mainly in Ecuador, awareness of ChatGPT was moderate and actual use was limited; however, many respondents expressed cautious optimism about its potential utility. Positive attitudes were more common among respondents with greater self-reported knowledge and higher ethical acceptance, whereas the strongest objections concerned AI-assisted ghostwriting and sole-source clinical use.

These findings support a practical next step. Health institutions should prioritize structured, context-specific training on privacy, data security, misinformation, authorship transparency, and human responsibility in clinical decisions before large-scale integration of generative AI tools is pursued. More regionally representative studies are needed to determine how these patterns vary across Latin American health systems.

## Data Availability

The original contributions presented in the study are included in the article/Supplementary Material, further inquiries can be directed to the corresponding author.
